# Antimicrobial resistance profile of *Pseudomonas aeruginosa* clinical isolates from healthcare-associated infections in Ethiopia: A systematic review and meta-analysis

**DOI:** 10.1371/journal.pone.0308946

**Published:** 2024-08-13

**Authors:** Zelalem Asmare, Melese Abate Reta, Yalewayker Gashaw, Ermias Getachew, Assefa Sisay, Muluken Gashaw, Ephrem Tamrat, Atitegeb Abera Kidie, Wagaw Abebe, Tadesse Misganaw, Agenagnew Ashagre, Zelalem Dejazmach, Getinet Kumie, Marye Nigatie, Sisay Ayana, Abdu Jemal, Solomon Gedfie, Woldeteklehaymanot Kassahun, Mulat Awoke Kassa, Selamyhun Tadesse, Biruk Beletew Abate

**Affiliations:** 1 Department of Medical Laboratory Science, College of Health Sciences, Woldia University, Woldia, Ethiopia; 2 Department of Medical Microbiology, Faculty of Health Sciences, University of Pretoria, Pretoria, South Africa; 3 Department of Public Health, College of Health Sciences, Woldia University, Woldia, Ethiopia; 4 Department of Nursing, College of Health Sciences, Woldia University, Woldia, Ethiopia; Nova Southeastern University, UNITED STATES OF AMERICA

## Abstract

**Background:**

Antimicrobial-resistant (AMR) bacterial infection is a significant global threat to the healthcare systems. *Pseudomonas aeruginosa*, the leading infectious agent in the healthcare setting is now one of the major threats due to AMR. A comprehensive understanding of the magnitude of AMR, particularly highly public health important pathogens such as *P*. *aeruginosa*, is necessary for the management of infections based on local information.

**Objective:**

This systematic review and meta-analysis aimed to determine the country-wide AMR of *P*. *aeruginosa*.

**Methods:**

Systematic searches were performed to retrieve articles from PubMed, Scopus, Web of Science, ScienceDirect electronic databases, Google Scholar search engine, and repository registrars from 2015 to 31^st^ December 2023. Twenty-three studies that provided important data on AMR in *P*. *aeruginosa* were systematically reviewed and analyzed to determine the country-wide magnitude of *P*. *aeruginosa* AMR profile from healthcare-associated infections. AMR of *P*. *aeruginosa* to 10 different antibiotics were extracted separately into Microsoft Excel and analyzed using STATA 17.0. Cohen’s kappa was computed to determine the agreement between reviewers, the Inverse of variance (I^2^) was used to evaluate heterogeneity across studies, and Egger’s test to identify publication bias. A random effect model was used to determine the pooled resistance to each antibiotic. Subgroup analysis was performed by infection type and year of publication.

**Results:**

This systematic review and meta-analysis revealed that the pooled prevalence of *P*. *aeruginosa* in clinical specimens associated with HAI was 4.38%(95%CI: 3.00–5.76). The pooled prevalence of AMR in *P*. *aeruginosa* for different antibiotics varies, ranging from 20.9% (95%CI: 6.2–35.8) for amikacin to 98.72% (95%CI: 96.39–101.4) for ceftriaxone. The pooled resistance was higher for ceftriaxone (98.72%), Trimethoprim-sulfamethoxazole (75.41), and amoxicillin-clavulanic acid (91.2). In contrast relatively lower AMR were observed for amikacin (20.9%) and meropenem (28.64%). The pooled multi-drug resistance (MDR) in *P*. *aeruginosa* was 80.5% (95%CI: 66.25–93.84). Upon subgroup analysis by infection types and year of publication, *P*. *aeruginosa* isolated from healthcare-associated infections exhibited higher resistance to ceftazidime (94.72%) compared to isolates from mixed types of healthcare-associated infections (70.84%) and surgical site infections (57.84%). Antimicrobial resistance in gentamicin was higher during the periods of 2018–2020 (73.96%), while comparatively lower during 2021–2023 (42.69%) and 2015–2017 (29.82%)

**Conclusions:**

Significantly high AMR and MDR were observed from this systematic review and meta-analysis. AMR obtained from this systematic review and meta-analysis urges the need for improved infection control, antimicrobial stewardship practices, and strengthened surveillance systems to control the spread of AMR and ensure effective treatment of P. aeruginosa infections.

**Protocol registration:**

This systematic review and meta-analysis was registered on PROSPERO (Registration ID: CRD42024518145).

## Introduction

Healthcare-associated infections (HAIs) are defined as infections that an individual acquires during medical treatment for other conditions [1]. *Pseudomonas aeruginosa* (*P*. *aeruginosa*) is a ubiquitous Gram-negative bacterium with simple nutritional requirements that exhibit the ability to thrive in various environments, including water, surfaces, medical devices, and hospital waste products [2]. *P*. *aeruginosa* is one of the prominent opportunistic pathogens, contributing to HAIs such as pneumonia, bloodstream infections, surgical site infections (SSI), urinary tract infections (UTI), burn wound infections, keratitis, and otitis media [3].

Infectious diseases resulting from antimicrobial-resistant (AMR) bacteria pose a significant global threat to healthcare systems, with an estimated 4.95 million global deaths associated with bacterial AMR in 2019, including 1.27 million deaths directly attributable to bacterial AMR [4]. *P*. *aeruginosa* was one of the six major bacterial pathogens (*E*. *coli*, *S*. *aureus*, *K*. *pneumoniae*, *S*. *pneumoniae*, *A*. *baumannii*, and *P*. *aeruginosa*) responsible for 18.8% of all deaths associated with AMR globally [4]. In the World Health Organization (WHO) African region, an estimated 1.05 million deaths were associated with bacterial AMR, from this, 250, 000 deaths were directly attributable to bacterial AMR in 2019 [5]. Apart from its impacts on mortality and disability, AMR also incurs substantial economic burdens. According to the estimates by the World Bank, AMR could lead to an extra US$1 trillion in healthcare expenses by 2050. Additionally, it could cause annual gross domestic product losses ranging from US$1 trillion to US$3.4 trillion by 2030 [6].

The misuse and overuse of antimicrobials in diverse sectors drive the rise of AMR pathogens, impacting nations of all income levels, especially worsening conditions in low- and middle-income countries (LMICs) [7]. Factors such as inappropriate antimicrobial use, easy access without prescription, lack of public awareness of proper usage, and inadequate surveillance systems exacerbate the prevalence of infections caused by AMR pathogens, particularly in developing nations [8].

Antimicrobial-resistant *P*. *aeruginosa* has been identified as a critical priority pathogen by the WHO [9]. *P*. *aeruginosa* has developed multi-drug resistance (MDR) by modifying outer membrane permeability, utilizing efflux pumps, producing antibiotic-inactivating enzymes, and facilitating the transfer of resistance genes or undergoing mutation, making the treatment of common infectious diseases challenging [10].

Understanding the extent and severity of AMR in *P*. *aeruginosa* is imperative, given its significance as a prominent opportunistic bacterium causing HAI. Previous studies conducted in Ethiopia have assessed the prevalence of AMR in *P*. *aeruginosa* against various antibiotics. However, the findings have been inconsistent, with reported resistance rates to different antibiotics ranging from zero [11] to one hundred percent [12–15] across different studies. In Ethiopia, there is a lack of systematic review and meta-analysis on *P*. *aeruginosa*, a predominant healthcare-associated pathogen. Therefore this systematic review and meta-analysis were undertaken to assess the comprehensive AMR profile of *P*. *aeruginosa*, which will provide crucial insights for guiding empirical therapy, infection control measures, and antibiotic stewardship efforts in Ethiopian healthcare settings. Furthermore, beyond its local significance, understanding the magnitude of AMR in P. aeruginosa will contribute to global health initiatives aimed at combating antimicrobial resistance, particularly against multidrug-resistant pathogens.

## Methods

### Protocol registration

This systematic review and meta-analysis have been registered on PROSPERO (International Prospective Register of Systematic Reviews) (registration ID: CRD42024518145).

### Databases and search strategy

Systematic searches were conducted across various databases, including PubMed, Scopus, Web of Science, and ScienceDirect electronic databases, to retrieve published articles. Additionally, articles available on Google Scholar and online repository sites of different institutions were also retrieved as part of the search process. Appropriate MeSH (Medical Subject Headings) terms and keywords were employed to retrieve relevant articles published in the English language within the timeframe of January 1, 2015, to December 31, 2023, from the listed databases. The search terms were: (((Antimicrobial resistance [MeSH Terms]) OR (Antibiotic resistance [MeSH Terms]) OR (Microbial drug resistance [MeSH Terms])) AND *Pseudomonas aeruginosa* [MeSH Terms] AND (Nosocomial infection) OR (Hospital-acquired infection) OR (Healthcare-associated infection)) AND Ethiopia. The complete search strategy and searching strings for different databases are depicted in the (**S1 Table in S1 File**). Furthermore, we reviewed the reference lists of primary studies and review papers to identify grey literature.

### Eligibility criteria

**Fig 1:**
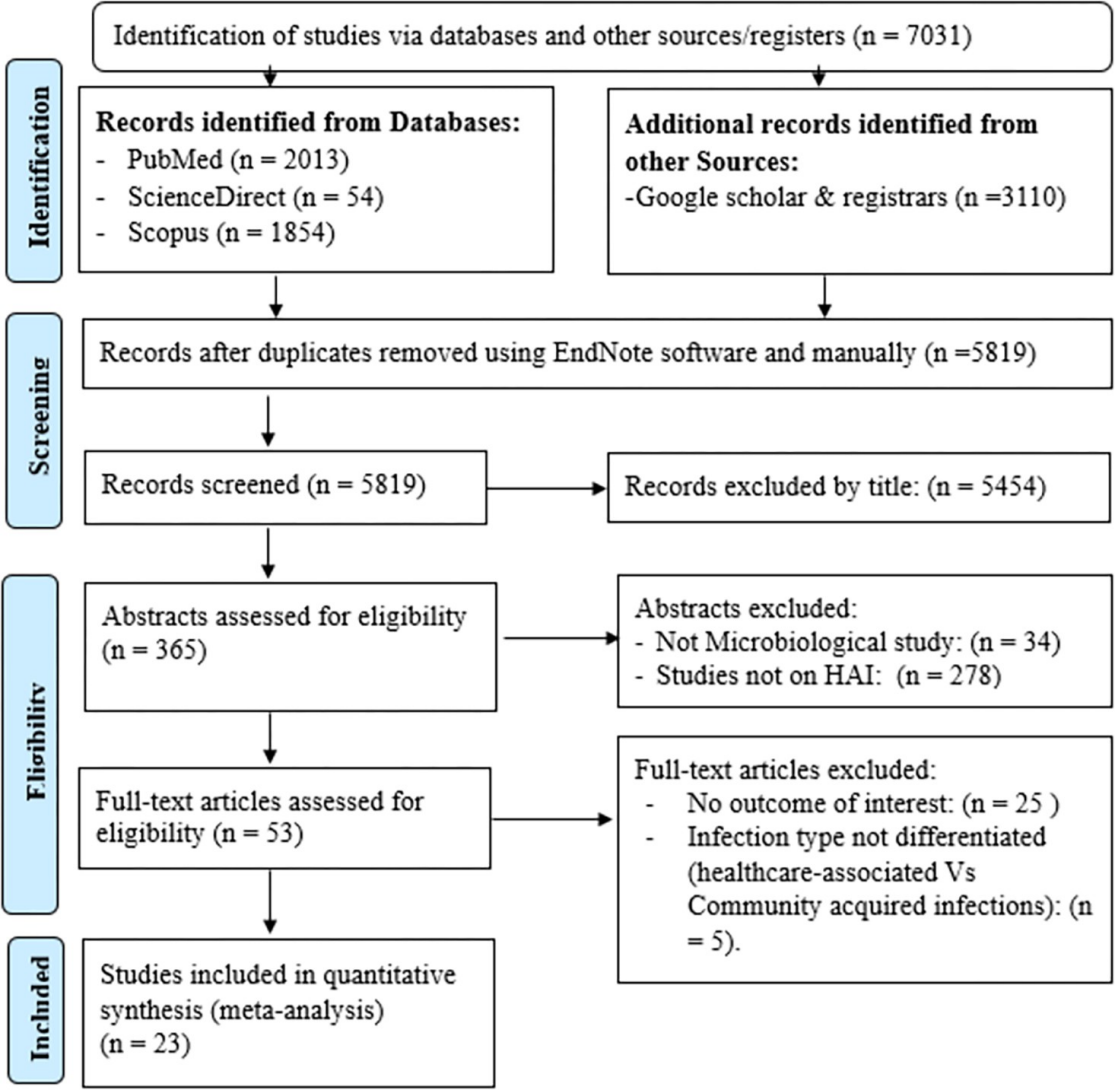
PRISMA flow diagram to show the result of the search and the reason for exclusion.

Studies obtained from the aforementioned databases were imported into EndNote X7 reference management software (Thomson Reuters, Toronto, Ontario, Canada), and following the updated Preferred Reporting Items for Systematic Reviews and Meta-Analyses (PRISMA) [16] (**Fig 1**), duplicates were eliminated, and the remaining studies underwent initial screening by titles, followed by detailed abstract and full-text screening by two reviewers (ZA and EG). To identify eligible articles, we employed predetermined inclusion and exclusion criteria. The inclusion criteria comprised; (a) articles published exclusively in the English language, (b) studies that reported the proportion or percentage of AMR in *P*. *aeruginosa* utilizing appropriate phenotypic or molecular AMR detection methods, and (c) studies focused solely on HAI or clinical samples. Studies that did not adhere to the aforementioned inclusion criteria were excluded. Moreover, studies that presented combined AMR results in categories such as "Gram-negative bacteria" and "Others," lacked explicit information regarding whether the infection type was hospital-acquired or community-acquired, as well as studies reported both hospital-acquired and community-acquired infections without differentiating the types of isolates and their respective AMR patterns, studies that didn’t report the outcomes of interest were excluded.

### Quality assessments

To assess the quality of each study, we utilized the Joanna Briggs Institute tool designed for prevalence and cohort studies [17]. Two impartial and independent reviewers (ZA and MAR) conducted a critical appraisal of each study. In instances where consensus between the two independent reviewers could not be achieved, a third reviewer (YG) was enlisted to resolve any disagreements and reach on consensus. Studies with a final quality score of 50% or higher were considered for inclusion in the systematic review and meta-analysis (**S2 Table in S1 File**).

### Data extraction

A standardized data extraction form on Microsoft Excel 2016 was utilized to systematically gather or record relevant information from each included potential study. The extraction process covered various domains, including study characteristics, such as the name of the author(s), publication year, study design, geographic location, types of participants (patients undergo surgery, catheterized patients, all-age patients, pediatric patients, post-partum and post-abortion women) and the number of study participants, clinical data, such as infection type (Healthcare-associated urinary tract infection (HAUTI), SSI, mixed type of HAI, puerperal sepsis) and specimen type (swab/pus, cerebrospinal fluid, blood, urine), the total number of bacteria isolates, and the number of *P*. *aeruginosa isolate*. It covered details on the AMR profile of *P*. *aeruginosa* to various antibiotics, including the number of tested isolates, the count of AMR isolates (at least for one antibiotic), and the number of MDR *P*. *aeruginosa* isolates (**Table 1**).

**Table 1:** Summary of included studies in systematic review and meta-analysis on antimicrobial-resistant *P*. *aeruginosa* healthcare-associated infections.

S.N	Author/s	Year of publication	Study region	Study design	Sample size	No of total bacterial isolates	No of *P*. *aeruginosa* isolates and tested for AMR	No of P. aeruginosa resistance to at least one antibiotic	Reports
**1**	Abayneh et al	2022	SNNPR	CH	262	41	5	4	AMR
**2**	Abosse et al	2021	AM	CS	165	115	26	22	AMR, MDR
**3**	Adugna et al	2021	AM	CS	422	53	3	2	AMR
**4**	Dagninet et al	2022	SNNPR	CS	245	72	10	7	AMR, MDR
**5**	Alemayehu et al	2019	SNNPR	CS	384	47	4	4	AMR, MDR
**6**	Ali et al	2023	AM	CS	338	48	2	2	AMR, MDR
**7**	Asmare et al	2023	AM	CS	211	52	6	6	AMR, MDR
**8**	Asres et al	2017	AA	CS	197	168	9	8	AMR, MDR
**9**	Awoke et al	2019	OR	CS	143	60	3	3	AMR
**10**	Bekele et al	2015	OR	CS	73	36	36	0	AMR
**11**	Bizuayehu et al	2022	AA	CS	220	79	12	10	AMR, MDR
**12**	Dessie et al	2016	AA	CS	107	104	6	6	AMR, MDR
**13**	Gashaw et al	2018	OR	CS	240	126	9	9	AMR, MDR
**14**	Gebissa et al	2021	OR	CS	150	147	18	16	AMR
**15**	Mekonen et al	2021	AM	CS	254	34	18	15	AMR, MDR
**16**	Melaku et al	2023	DD	CS	188	120	9	9	AMR
**17**	Misha et al	2021	OR	CH	251	38	8	8	AMR
**18**	Motbinor et al	2020	AM	CS	238	20	11	11	AMR, MDR
**19**	Sahile et al	2016	OR	CS	200	111	8	7	AMR, MDR
**20**	Tilahun et al	2022	AM	CS	384	343	31	18	AMR, MDR
**21**	Tilahun et al	2022	AM)	CS	423	75	46	25	AMR, MDR
**22**	Tolera et al	2018	HR	CS	394	54	6	5	AMR
**23**	Worku et al	2023	Mixed	CS	752	494	18	12	AMR, MDR

Abbreviation: AM: Amhara; AA: Addis Ababa, OR: Oromia; SNNPR: South Nation and Nationality and Peoples Regions; HR: Harari; DD: Dire Dawa: AMR: Antimicrobial resistance; MDR: Multidrug resistance; CH: cohort; CS: cross-sectional

### Statistical analysis

The data was initially entered into a prepared Microsoft Excel sheet, and subsequently, it was exported to STATA 17.0 software (StataCorp, Texas, USA) for final analysis. The inverse variance (I^2^) test was used to assess the heterogeneity across studies with interpretations assigned to I^2^ values: 0% (no heterogeneity), 0–25% (low heterogeneity), 25–50% (medium heterogeneity), and >75% (high heterogeneity) [18]. A subgroup analysis based on various categories was performed for studies that exhibited high heterogeneity. The Egger’s test was employed to evaluate the presence of publication bias with a significance threshold of *p* < 0.05, and a trim-and-fill analysis was conducted to address and manage potential bias. A random effect model for meta-analysis was used to estimate the pooled prevalence of *P*. *aeruginosa* in clinical specimens associated with HAI, and the pooled prevalence of AMR and MDR *P*. *aeruginosa*. The aggregate prevalence of HAI associated with *P*. *aeruginosa* was determined by assessing the proportion of *P*. *aeruginosa* cases among the total number of specimens. To calculate the pooled prevalence of AMR and MDR continuity correction was made for zero and one-hundred percent AMR values which resulted in zero standard error [19]. Finally, the pooled prevalence of AMR was calculated separately for each antibiotic tested.

## Results

### A descriptive summary of included studies

This systematic review and meta-analysis encompassed 23 studies that provided important data on the microbiologically confirmed prevalence, AMR, and MDR profile of *P*. *aeruginosa* isolates. In this review, 6,212 study participants suspected of hospital-acquired infections were assessed for bacterial infections, yielding a total of 2,437 hospital-acquired infections. From these studies, 304 isolates of *P*. *aeruginosa* were obtained and tested for resistance to a maximum of ten different antibiotics. All the studies included followed Clinical Laboratory Standard Institute (CLSI) guidelines to report AMR resistances and considered bacterial isolates as MDR based on the published guideline by Magiorakos, et al. [20]. In these studies, the prevalence of *P*. *aeruginosa* associated with HAI ranged from 0.59–15.76% [11–15, 21–38] (**Table 1**).

### The pooled prevalence of *P*. *aeruginosa* in healthcare-associated infections

**Fig 2:**
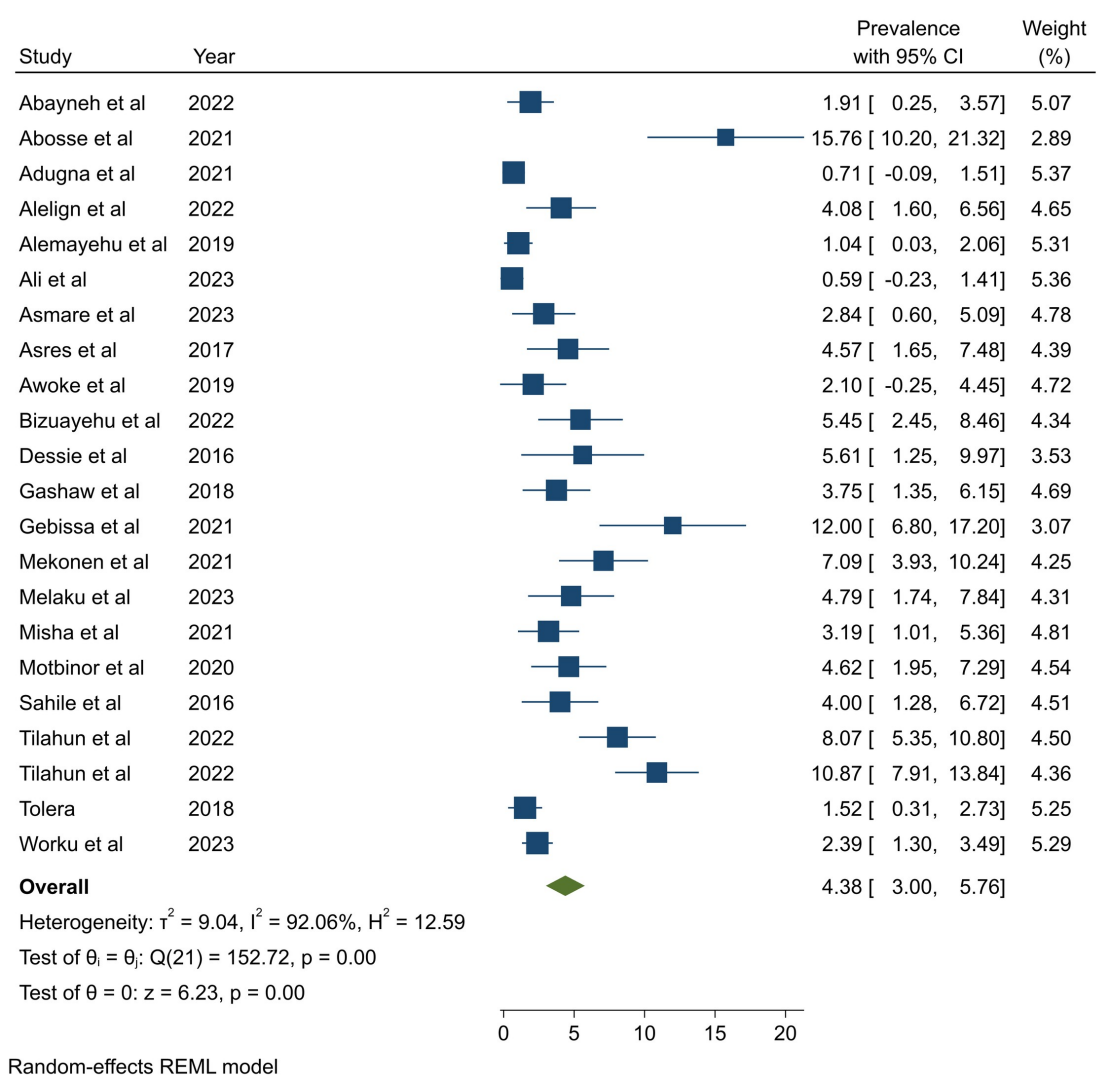
Forest plot showing the prevalence of *P*. *aeruginosa* associated with healthcare-associated infections.

In this systematic review and meta-analysis, the pooled prevalence of *P*. *aeruginosa* associated with HAI was determined to be 4.38% (95%CI: 3.00–5.76) (**Fig 2**). Since Egger’s test revealed the presence of publication bias (P <0.001), to correct the publication bias, the trim-and-fill analysis was performed, and the pooled prevalence was found to be 4.61% (95%CI: 3.23–6.00) (**S3 Table in S1 File**). Even if high heterogeneity (I^2^ = 92.06) was observed across studies, subgroup analysis by types of infection and year of publication showed no significant variation in the prevalence of *P*. *aeruginosa* associated HAI (**S10 and S11 Figs in S1 File**).

**Fig 3:**
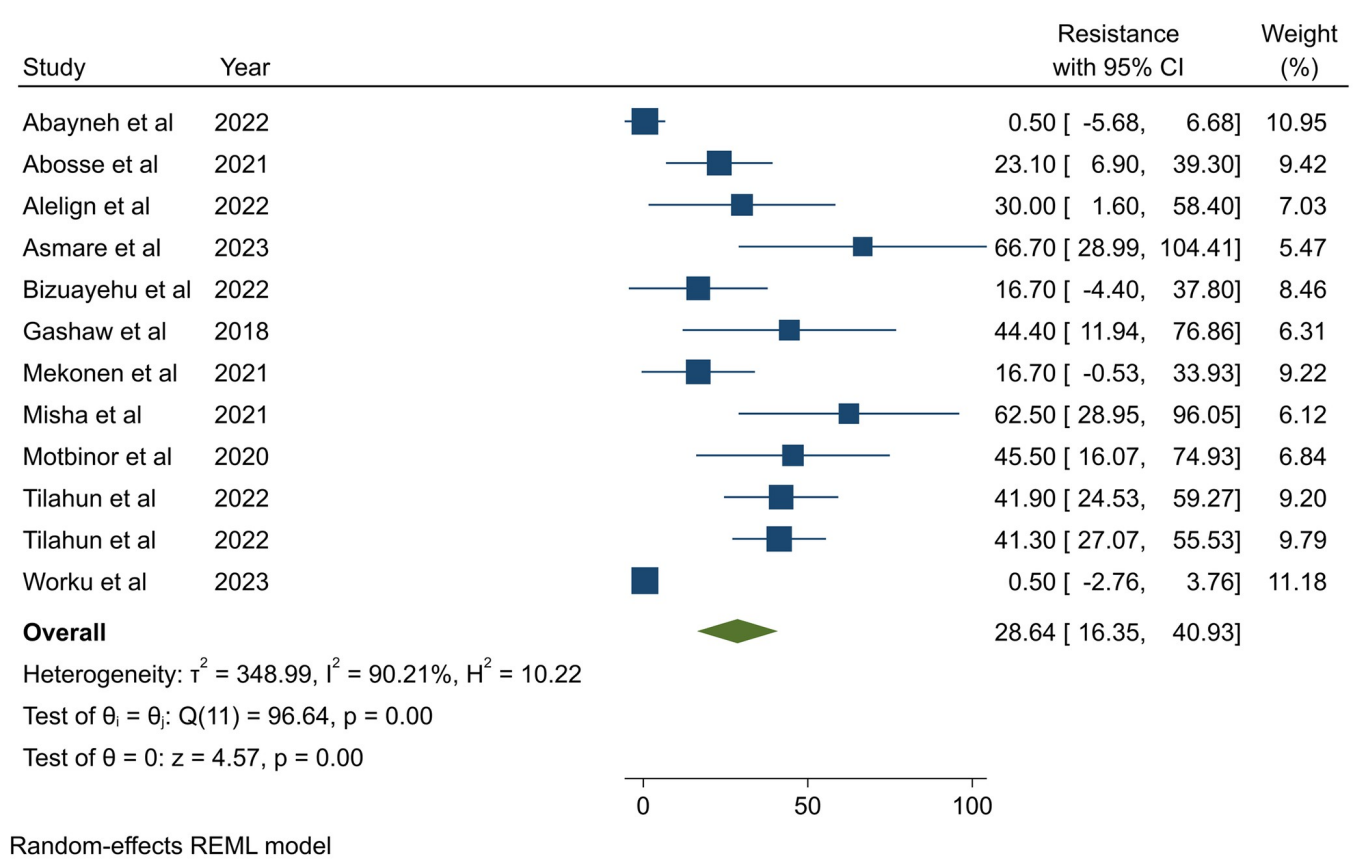
Pooled antimicrobial resistance of *P*. *aeruginosa* to meropenem.

**Antimicrobial resistance in *P*. *aeruginosa*.** The pooled prevalence of AMR in *P*. *aeruginosa* was calculated based on a maximum of 22 studies for gentamicin [11–15, 21–24, 26, 27, 29–38] and ciprofloxacin [11–15, 21–24, 26, 27, 29–38] and a minimum of 7 studies for trimethoprime-sulfamethoxazole [13–15, 26–28, 33], with a total of 10 antibiotics (amikacin, amoxicillin-clavulanic acid, ampicillin, ceftazidime, ceftriaxone, chloramphenicol, ciprofloxacin, trimethoprim-sulfamethoxazole, gentamicin, and meropenem) being pooled separately (**Table 2**). The pooled prevalence of AMR in *P*. *aeruginosa* for the listed antibiotics varies, ranging from 20.9% (95%CI: 6.2–35.8) for amikacin to 98.72% (95%CI: 96.39–101.4) for ceftriaxone (**Table 2**). From this systematic review and meta-analysis, it was found that the pooled AMR of *P*. *aeruginosa* to third-generation cephalosporins was higher, ranging from 66.8% for ceftazidime to 98.72% for ceftriaxone. In contrast, relatively lower levels of AMR were observed for amikacin (20.9%) and the last resort antibiotics, carbapenem/meropenem (28.64%) (95%CI: 16.35–40.93) (**Fig 3**). The Egger’s test showed that there was publication bias across studies used to estimate the pooled resistance of meropenem, ceftriaxone, amoxicillin-clavulanic acid, and trimethoprim-sulfamethoxazole (**Table 2**). To address the publication bias trim-and-fill analysis was computed and resulted in a significant change in AMR of meropenem, ceftriaxone, and trimethoprime-sulfamethoxazole (**S4-S6 Tables in S1 File**). For amoxicillin-clavulanic acid, there was no effect on the AMR of antibiotics after trim-and-fill analysis.

**Table 2:** The pooled prevalence of *Pseudomonas aeruginosa* to ten different antibiotics.

Antibiotics	No of studies	Pooled resistance (95% CI)	Pooled resistance after trim-and-fill analysis	Heterogeneity (I^2^) (*p*-value)	(Egger’s test) *p*-value
**Amikacin**	8	20.98 (6.2–35.8)		92.17% (<0.01)	0.166
**Amoxicillin-clavulanic acid**	8**	91.2 (80.6–101.8)	No change	92.48% (<0.01)	<0.001
**Ampicillin**	9	79.66 (56.6–102.8)		99.07% (<0.01)	0.210
**Ceftazidime**	17	66.85 (54.6–79.1)		91.04% (<0.01)	0. 209
**Ceftriaxone**	13 (3*)	98.72 (96.39–101.04)	99.1 (96.8–101.4)	0.01% (0.13)	<0.001
**Chloramphenicol**	9	69.2 (52.8–85.6)		82.52% (<0.01)	0.122
**Ciprofloxacin**	22	46.5 (35.3–57.7)		90.75% (<0.01)	0.434
**Trimethoprim-Sulfamethoxazole**	11 (4*)	75.41 (58.39–92.43)	92.1 (72.9–111.3)	70.52% (<0.01)	<0.001
**Gentamicin**	22	47.4 (35.3–59.5)		93.46% (<0.01)	0.317
**Meropenem**	14 (2*)	28.64 (16.35–40.93)	24.1 (11.5–36.7)	90.21% (<0.01)	<0.001

* The number of imputed studies during Trim-and-fill analysis

** No effect on the pooled prevalence after Trim-and-fill analysis; CI: Confidence Interval; I^2^: Inverse of Variance. **Forest plots** of the pooled resistance of *P*. *aeruginosa* for each antibiotic are available in **(S1-S9 Figs in S1 File**)

Inverse of variance (I^2^) statistics showed greater than 70.0% heterogeneity among studies for all antibiotics except studies pooled to estimate the resistance of ceftriaxone (**Table 2**). To identify the possible source of heterogeneity, subgroup analysis was performed for each antibiotic by year of publication and type of infection (**S12-S27 Figs in S1 File)**. Subgroup analysis based on study year and infection type revealed noteworthy disparities in the AMR of ceftazidime and gentamicin. Particularly, when examining infection types, it was evident that *P*. *aeruginosa* isolated from HAUTI exhibited higher resistance to ceftazidime (94.72%) compared to isolates from mixed types of HAI (70.84%) and SSI (57.84%) (**S15 Fig in S1 File**). Notable fluctuations in gentamicin AMR were observed across different years, with resistance rates being higher during the periods of 2018–2020 (73.96%), while comparatively lower during 2021–2023 (42.69%) and 2015–2017 (29.82%) (**S24 Fig in S1 File**).

### Multi-drug resistance profile of *P*. *aeruginosa*

**Fig 4:**
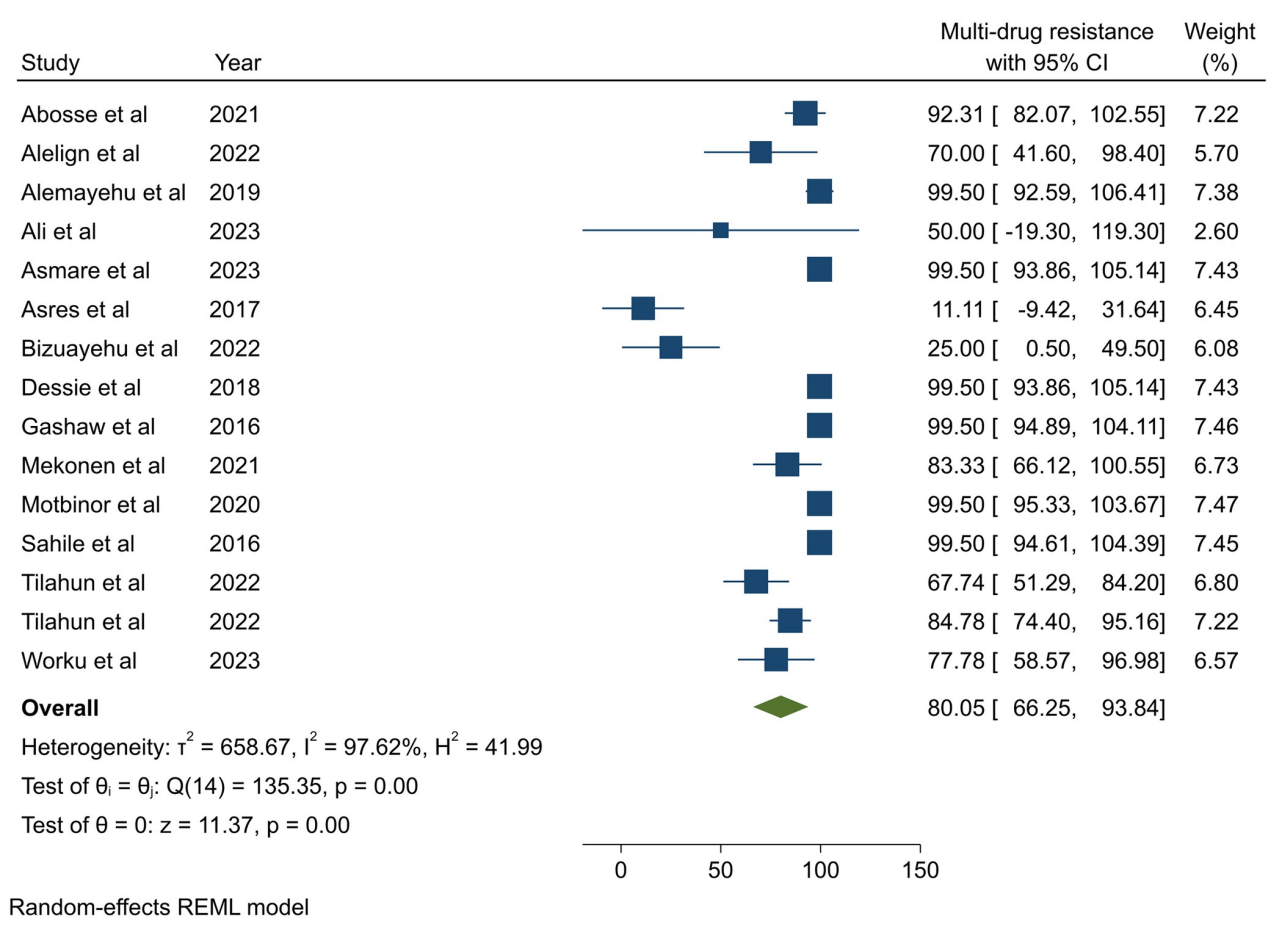
Pooled multi-drug resistance profile of *P*. *aeruginosa* isolated from healthcare-associated infections.

**Fig 5:**
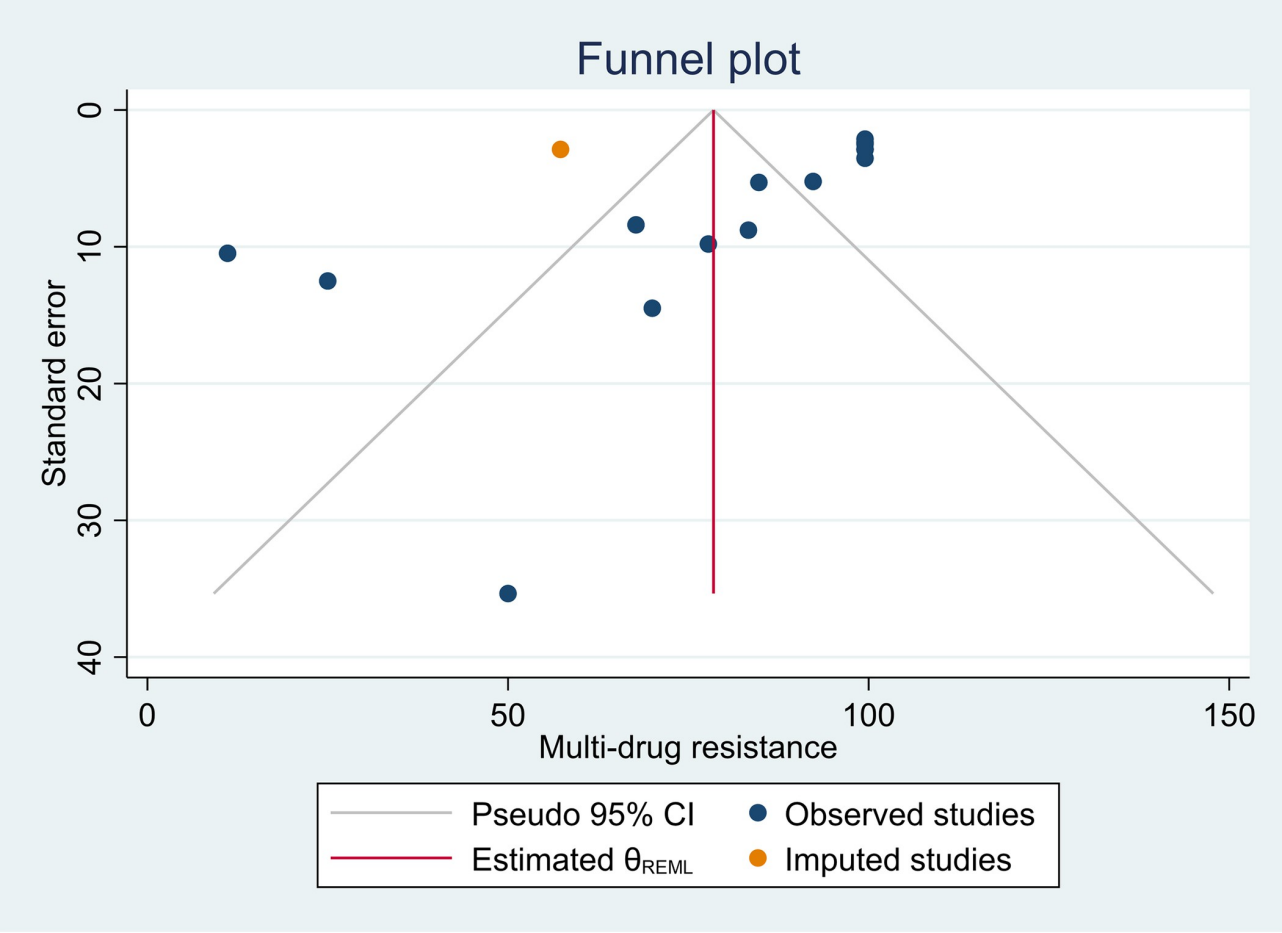
Funnel plot for multi-drug resistance of *P*. *aeruginosa* after trim-and-fill analysis.

**Fig 6:**
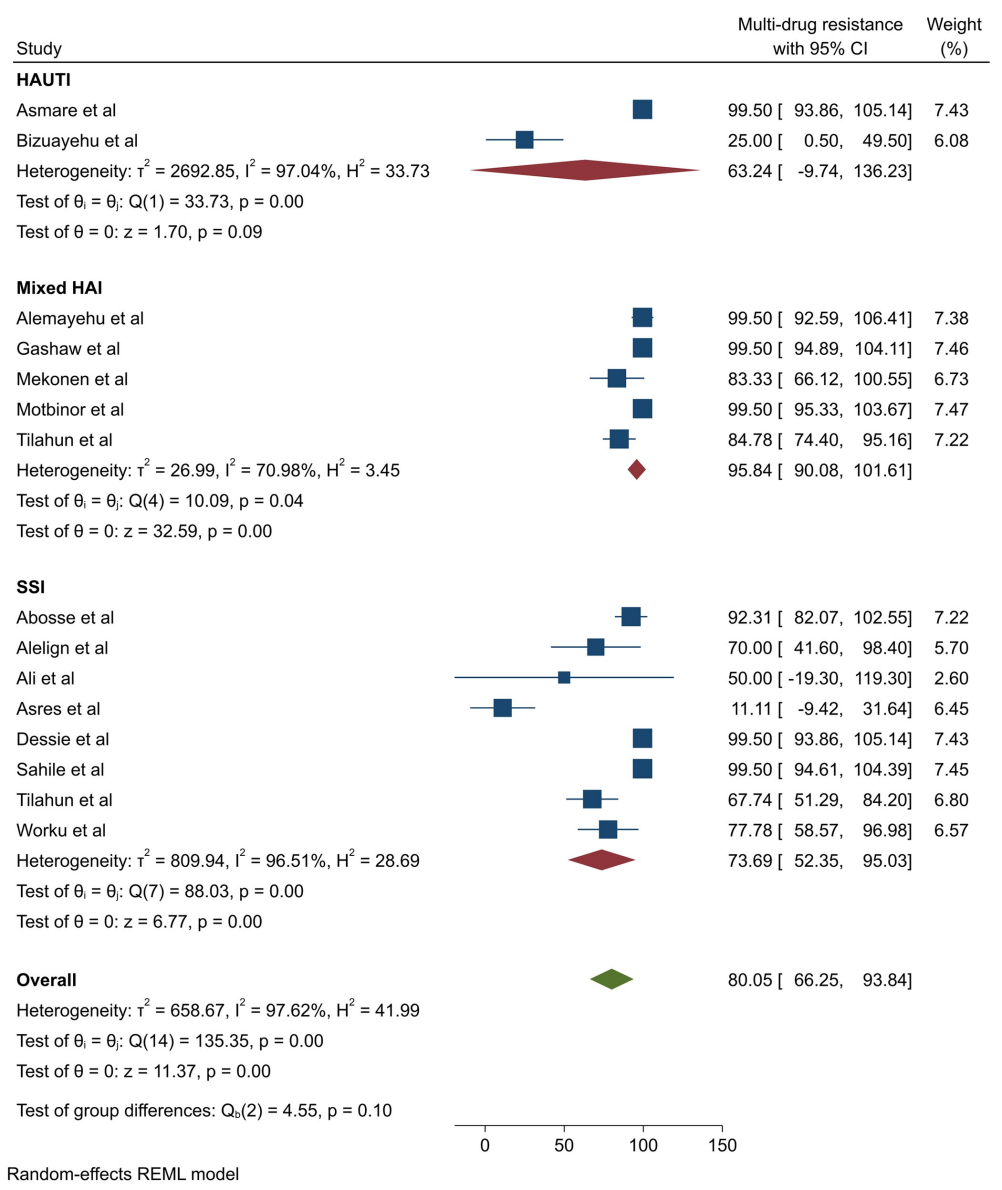
Subgroup analysis of multi-drug resistant *P*. *aeruginosa* isolated from healthcare-associated infections by infection type.

The pooled prevalence of MDR in *P*. *aeruginosa* was 80.05% (95%CI: 66.25–93.84) (**Fig 4**). However, Egger’s test revealed the presence of publication bias and was subjected to trim-and-fill analysis, and the pooled MDR of *P*. *aeruginosa* was adjusted to be 78.49% (95%: CI 65.27–91.72) (**S7 Table in S1 File and Fig 5**). High heterogeneity, indicated by an I^2^ value of 97.62%, was noted across studies. Subsequently, subgroup analysis was conducted based on infection types and publication years. The analysis revealed a higher prevalence of MDR cases among mixed types of HAI (95.84%) compared to SSI (73.69%) and HAUTI (63.24%) (**Fig 6**). However, subgroup analysis based on years of publication did not demonstrate significant variation (**S28 Fig in S1 File**).

## Discussion

The findings of this systematic review and meta-analysis provide a comprehensive insight into the alarming rates of AMR observed in *P*. *aeruginosa* isolates from HAIs within Ethiopian healthcare settings. Our analysis reveals a concerning burden, with AMR prevalence ranging from 20.9% to 98.72% across ten different antibiotics analyzed. This wide spectrum of resistance underscores the complexity and severity of the AMR crisis facing healthcare facilities in Ethiopia. Moreover, the high prevalence of MDR *P*. *aeruginosa* (80.0%), poses a substantial challenge to the effective management and treatment of HAIs. Notably, our findings also indicate that 4.38% of HAIs in Ethiopian hospitals can be attributed to *P*. *aeruginosa*. These results highlight the urgent need for targeted interventions and strengthened antimicrobial stewardship programs to combat the spread of AMR and mitigate its impact on patient outcomes and healthcare delivery in Ethiopia.

In this thorough systematic review and meta-analysis conducted in Ethiopia, *P*. *aeruginosa* was identified as responsible for 4.38% of HAIs, a prevalence rate consistent with findings reported from China (6.53%) [39]. However, this prevalence is lower than the reported rate from Egypt (19.9%) [40]. Variations in prevalence rates of *P*. *aeruginosa* HAIs across regions can be attributed to differences in healthcare practices, antibiotic usage, environmental factors, healthcare infrastructure, and population characteristics.

The AMR pattern of *P*. *aeruginosa* for aminoglycoside antibiotics reveals varying resistance rates. The amikacin resistance rate of 20.9% in this study aligns with rates in Turkey (17.8%) [41], China (20.8%, 22.2%) [42, 43], and Somalia (20%) [44], while higher rates are seen in India (80%) [45] and Nepal (37.5%) [46]. Additionally, the gentamicin resistance rate of 47.4% in this study is in line with resistance rates in China (42.4%) [43] and Somalia (45.5%) [44], but exceeds rates in Turkey (28.2%) [41] and China (29.7%) [42]. Furthermore, lower resistance rates than in India (88%) [45] and Nepal (62.5%) [46] were observed. Factors including overuse of antibiotics, inadequate infection control practices, prolonged hospitalization, and limited surveillance in developing countries might be the possible reasons for this increased magnitude of AMR [47, 48].

*Pseudomonas aeruginosa* showed varying resistance rates to penicillin and cephalosporin antibiotics, which are among the most frequently prescribed antibiotics in Ethiopia. Specifically amoxicillin-clavulanic acid and ceftriaxone exhibited extremely high resistance levels (91.2%) and (98.72%), respectively. The amoxicillin-clavulanic acid resistance rate aligns with a report from Somalia (88.9%) [44] but surpasses rates reported in Iran (50.6%) [49]. Similarly, the ceftriaxone resistance rate exceeds rates reported from China (78.6%) [42]. Additionally, the ceftazidime resistance rate of 66.8%, although consistent with India (70%) [45] and Iran (57.75%) [50], surpasses rates in Turkey (38.6%) [41] and China (34.3%) [43], as well as a report from Somalia (53.8%) [44] and Spain (20.3%) [51]. However, lower ceftazidime resistance rates than in China (94.1%) and Nepal (91.6%) [46].

In this systematic review and meta-analysis, *P*. *aeruginosa* exhibited a noteworthy 28.64% resistance rate to meropenem, a critical last-resort antibiotic, indicating significant antimicrobial resistance against carbapenems. This resistance rate is comparable with resistance reported from Turkey (30.1%) [41], China (35.7%) [43], and Spain (14.1%) [51], although it exceeds a lower rate from China (7.7%) [42]. It is notably lower than reported resistance in Iran (40%) [49], Somalia (50%) [44], India (80%) [45], and Nepal (62.5%) [46].

The ciprofloxacin resistance rate of *P*. *aeruginosa* in this study, at 46.5%, aligns closely with rates reported in Iran (47.3%) [49] and Spain (38.4%) [51]. However, it surpasses rates in Turkey (30.7%) [41] and China (21.2% and 35%) [42, 43], as well as Somalia (14.3%) [44], though remaining lower than in India (96%) [45] and Nepal (95.8%) [46]. On the other hand, the Trimethoprim-Sulfamethoxazole resistance rate of 75.41% in this study indicated alarmingly high levels of resistance requiring immediate attention. This was consistent with the resistance rate in Somalia (89%) [44]. The variability of AMR rates of *P*. *aeruginosa* observed in our systematic review compared to studies abroad might be attributed to diverse local epidemiological factors, differences in antibiotic usage practices, variations in healthcare settings, and implementation of antibiotic stewardship programs [52, 53].

The MDR profile of *P*. *aeruginosa* isolated from HAIs in this comprehensive systematic review and meta-analysis was found to be 80.0%. This percentage aligns closely with MDR rates reported from Somalia (68%) [44] and Nepal (83.3%) [46]. However, it surpasses the rate reported from India (50%) [45], Spain (26.2%) [51], and Iran (58%) [49]. The difference in MDR *P*. *aeruginosa* across countries can be attributed to variations in antibiotic prescribing practices, AMR patterns, healthcare infrastructure, infection control measures, antibiotic stewardship programs, surveillance systems, and population characteristics such as prevalence of comorbidities and immunocompromised individuals.

Overall in this systematic review and meta-analysis, there was a significantly increased AMR in *P*. *aeruginosa* to different antibiotics which is an indicator of a lack of antimicrobial stewardship programs, surveillance of AMR, and infection prevention and control practices [48]. Clinicians in Ethiopian healthcare settings should reconsider empirical antibiotic therapy for healthcare-associated infections caused by *P*. *aeruginosa* due to high levels of antimicrobial resistance, necessitating adjustments in treatment protocols to optimize patient outcomes. Urgent implementation of antimicrobial stewardship programs is underscored by the study findings, which can promote judicious antibiotic use, optimize treatment regimens, and mitigate the spread of antimicrobial resistance among *P*. *aeruginosa* isolates. Strengthening infection control practices, including improved hand hygiene, environmental disinfection, and patient isolation protocols, is crucial to prevent and contain the spread of antimicrobial-resistant *P*. *aeruginosa* within Ethiopian healthcare settings. Moreover, the study provides valuable data for informing policy decisions aimed at addressing antimicrobial resistance in Ethiopia and facilitating the development of evidence-based strategies for antimicrobial stewardship, infection prevention, surveillance, and resource allocation to combat the growing threat of antimicrobial resistance in healthcare settings.

Future research could focus on investigating novel therapeutic strategies, exploring the molecular mechanisms underlying antimicrobial resistance in *P*. *aeruginosa*, assessing the impact of local epidemiological factors, evaluating interventions to reduce resistance prevalence, and assessing the impact of socioeconomic factors on antimicrobial resistance dynamics within healthcare settings to improve patient care.

### Strength and limitations

This systematic review and meta-analysis have strengths such as employing a predefined protocol for search strategy and data extraction, alongside internationally recognized tools for critical appraisal to evaluate study quality. However, limitations were observed due to the inclusion criteria restricting studies solely published in English and selection bias. The included studies varied in quality, methodologies, and outcomes, contributing to heterogeneity in the results, despite our efforts to address this statistically. Publication bias is also a concern, as studies with positive findings are more likely to be published. Errors or inconsistencies in data extraction and analysis, although minimized, cannot be entirely eliminated. Additionally, inconsistencies in reporting specific data points across studies limited some subgroup analyses.

## Conclusion and recommendations

The findings of this systematic review and meta-analysis concerning the AMR profile of *P*. *aeruginosa* isolated from HAIs in Ethiopia revealed a significant prevalence of MDR, indicating a substantial challenge in managing infections caused by this pathogen in healthcare settings. The observed increase in AMR and MDR underscores the urgent need for enhanced infection control measures, careful antimicrobial stewardship practices, and strengthened surveillance systems to curb the spread of resistant strains and ensure effective treatment of *P*. *aeruginosa* infections in Ethiopia. Additionally, collaborative efforts at local, national, and international levels are warranted to address the multifaceted factors contributing to AMR and mitigate its impact on public health. Based on the finding from this comprehensive systematic review and meta-analysis we would like to recommend all stakeholders such as governmental, non-governmental organizations, health department officers, policy makers and researchers to work collaboratively to enhance infection prevention control and antimicrobial stewardship practices.

## Supporting information

S1 Checklist PRISMA checklist for antimicrobial resistance of *P*. *aeruginosa* clinical isolates from healthcare-associated infections in Ethiopia. (DOCX)

S1 File Supporting tables and figures. (DOCX)

## Abbreviations

AMR: Antimicrobial Resistance
HAI: Healthcare-associated Infection
HAUTI: Healthcare-associated Urinary Tract Infection
MDR: Multi-drug Resistance
SSI: Surgical Site Infection
