# Supplementary material for: Antimicrobial resistance profile of Pseudomonas aeruginosa clinical isolates from healthcare-associated infections in Ethiopia: A systematic review and meta-analysis
Source: PLoS One. 2024 Aug 13;19(8):e0308946. doi: 10.1371/journal.pone.0308946 (PMC11321567; doi:10.1371/journal.pone.0308946)
Supplement: S1 File — (DOCX) [file pone.0308946.s002.docx]

**Antimicrobial resistance profile of *Pseudomonas aeruginosa* isolated from healthcare-associated infections in Ethiopia. A Systematic Review and Meta-Analysis.**

*Supplementary file*

**List of figures and tables**

[**S1 Fig: Pooled antimicrobial resistance of P. aeruginosa for amikacin** 6](#_Toc173444204)

[**S2 Fig: Pooled antimicrobial resistance of P. aeruginosa for amoxicillin-clavulanic acid** 7](#_Toc173444205)

[**S3 Fig: Pooled antimicrobial resistance of P. aeruginosa to ampicillin** 8](#_Toc173444206)

[**S4 Fig: Pooled antimicrobial resistance of P. aeruginosa to ceftazidime** 9](#_Toc173444207)

[**S5 Fig: Pooled antimicrobial resistance of P. aeruginosa to ceftriaxone** 10](#_Toc173444208)

[**S6 Fig: Pooled antimicrobial resistance of P. aeruginosa to chloramphenicol** 11](#_Toc173444209)

[**S7 Fig: Pooled antimicrobial resistance of P. aeruginosa to gentamicin** 12](#_Toc173444210)

[**S8 Fig: Pooled antimicrobial resistance of P. aeruginosa to ciprofloxacin** 13](#_Toc173444211)

[**S9 Fig: Pooled antimicrobial resistance of P. aeruginosa to Trimethoprime-Sulfamethoxazole** 14](#_Toc173444212)

[**S10 Fig: Subgroup analysis of P. aeruginosa associated health-care associated infections by publication year** 15](#_Toc173444213)

[**S11 Fig: Subgroup analysis of P. aeruginosa associated healthcare-associated infections by infection type** 16](#_Toc173444214)

[**S12 Fig: Subgroup analysis of antimicrobial resistance of P. aeruginosa for amikacin by infection type** 17](#_Toc173444215)

[**S13 Fig: Subgroup analysis of antimicrobial resistance of P. aeruginosa for amoxicillin-clavulanic acid by infection type** 18](#_Toc173444216)

[**S14 Fig: Subgroup analysis of antimicrobial resistance of P. aeruginosa to ampicillin by infection type** 19](#_Toc173444217)

[**S15 Fig: Subgroup analysis of antimicrobial resistance of P. aeruginosa to ceftazidime by infection type** 20](#_Toc173444218)

[**S16 Fig: Subgroup analysis of antimicrobial resistance of P. aeruginosa to ceftazidime by publication year** 21](#_Toc173444219)

[**S17 Fig: Subgroup analysis of antimicrobial resistance of P. aeruginosa to ceftriaxone by infection type** 22](#_Toc173444220)

[**S18 Fig: Subgroup analysis of antimicrobial resistance of P. aeruginosa to ceftriaxone by publication year** 23](#_Toc173444221)

[**S19 Fig: Subgroup analysis of antimicrobial resistance of P. aeruginosa to chloramphenicol by infection type** 24](#_Toc173444222)

[**S20 Fig: Subgroup analysis of antimicrobial resistance of P. aeruginosa to chloramphenicol by publication year** 25](#_Toc173444223)

[**S21 Fig: Subgroup analysis of antimicrobial resistance of P. aeruginosa to ciprofloxacin by infection type** 26](#_Toc173444224)

[**S22 Fig: Subgroup analysis of antimicrobial resistance of P. aeruginosa to ciprofloxacin by publication year** 27](#_Toc173444225)

[**S23 Fig: Subgroup analysis of antimicrobial resistance of P. aeruginosa to gentamicin by Infection type** 28](#_Toc173444226)

[**S24 Fig: Subgroup analysis of antimicrobial resistance of P. aeruginosa to gentamicin by publication year** 29](#_Toc173444227)

[**S25 Fig: Subgroup analysis of antimicrobial resistance of P. aeruginosa to Trimethoprim-Sulfamethoxazole by type of infection** 30](#_Toc173444228)

[**S26 Fig: Subgroup analysis of antimicrobial resistance P. aeruginosa to meropenem by infection type** 31](#_Toc173444229)

[**S27 Fig: Subgroup analysis of antimicrobial resistance P. aeruginosa to meropenem by study year** 32](#_Toc173444230)

[**S28 Fig: Subgroup analysis of multi-drug resistant P. aeruginosa by publication year** 33](#_Toc173444231)

[**S29 Fig: Funnel plot for AMR of P. aeruginosa to Trimethoprim-Sulfamethoxazole after trim-and-fill analysis** 34](#_Toc173444232)

[**S30 Fig: Funnel plot for antimicrobial resistance of P. aeruginosa to ceftriaxone after trim-and-fill analysis** 35](#_Toc173444233)

[**S31 Fig: Funnel plot for antimicrobial resistance of P. aeruginosa to meropenem after trim-and-fill analysis** 36](#_Toc173444234)

**Tables**

**[S1 Table: Search strategy and searching strings for the PubMed database](#_Toc173444235)** [3](#_Toc173444235)

[**S2 Table: Quality appraisal result of included studies; Using Joanna Briggs Institute (JBI) quality appraisal checklist for prevalence studies.** 4](#_Toc173444236)

[**S3 Table: Trim-and-fill analysis for the prevalence of P. aeruginosa associated healthcare-associated infection** 34](#_Toc173444237)

[**S4 Table: Trim-and-fill analysis result of antimicrobial resistance of P. aeruginosa to Trimethoprim-Sulfamethoxazole** 34](#_Toc173444238)

[**S5 Table: Trim-and-fill analysis result of antimicrobial resistance of P. aeruginosa to ceftriaxone** 35](#_Toc173444239)

[**S6 Table: Trim-and-fill analysis result of antimicrobial resistance of P. aeruginosa to meropenem** 35](#_Toc173444240)

[**S7 Table: Trim-and-fill analysis on pooled multi-drug resistance profile of P. aeruginosa** 36](#_Toc173444241)

**S1 Table: Search strategy and searching strings for the PubMed database**

| Data bases | Search | | Number of articles |
| --- | --- | --- | --- |
| PubMed | Search: **(((Antimicrobial resistance [MeSH Terms]) OR (Antibiotic resistance [MeSH Terms]) OR (Microbial drug resistance [MeSH Terms])) AND Pseudomonas aeruginosa [MeSH Terms] AND (Nosocomial infection) OR (Hospital-acquired infection) OR (Healthcare-associated infection)) AND Ethiopia.** Filters: **English, from 2015 - 2023** | (((("drug resistance, microbial"[MeSH Terms] OR "drug resistance, microbial"[MeSH Terms] OR "drug resistance, microbial"[MeSH Terms]) AND "pseudomonas aeruginosa"[MeSH Terms] AND ("cross infection"[MeSH Terms] OR ("cross"[All Fields] AND "infection"[All Fields]) OR "cross infection"[All Fields] OR ("nosocomial"[All Fields] AND "infection"[All Fields]) OR "nosocomial infection"[All Fields])) OR ("cross infection"[MeSH Terms] OR ("cross"[All Fields] AND "infection"[All Fields]) OR "cross infection"[All Fields] OR ("hospital"[All Fields] AND "acquired"[All Fields] AND "infection"[All Fields]) OR "hospital acquired infection"[All Fields]) OR ("cross infection"[MeSH Terms] OR ("cross"[All Fields] AND "infection"[All Fields]) OR "cross infection"[All Fields] OR ("healthcare"[All Fields] AND "associated"[All Fields] AND "infection"[All Fields]) OR "healthcare associated infection"[All Fields])) AND ("ethiopia"[MeSH Terms] OR "ethiopia"[All Fields] OR "ethiopia s"[All Fields])) AND ((english[Filter]) AND (2015:2023[pdat])) | 2013 |

**S2 Table: Quality appraisal result of included studies; Using Joanna Briggs Institute (JBI) quality appraisal checklist for prevalence studies.**

| Authors | **1)** **Appropriate sample frame** | **2) Appropriate sampling** | **3) adequacy of sample size** | **4) study subjects and setting described in detail** | **5) data analysis conducted with sufficient coverage of the identified sample** | **6) valid methods for the identification of the condition** | **7) condition measured in a standard, reliable way** | **8) appropriate statistical analysis** | **9) adequacy of response rate** | **Total score/9** |
| --- | --- | --- | --- | --- | --- | --- | --- | --- | --- | --- |
| Abayneh et al | 0 | 1 | 1 | 1 | 1 | 1 | 1 | 1 | 1 | 8 |
| Abosse et al | 0 | 1 | 0 | 1 | 1 | 1 | 1 | 1 | 1 | 7 |
| Adugna et al | 1 | 1 | 1 | 1 | 1 | 1 | 1 | 1 | 1 | 9 |
| Dagninet et al | 0 | 1 | 1 | 1 | 1 | 1 | 1 | 1 | 1 | 8 |
| Alemayehu et al | 1 | 1 | 1 | 1 | 1 | 1 | 1 | 1 | 1 | 9 |
| Ali et al | 0 | 1 | 1 | 1 | 1 | 1 | 1 | 1 | 1 | 8 |
| Asmare et al | 0 | 1 | 1 | 1 | 1 | 1 | 1 | 1 | 1 | 8 |
| Asres et al | 0 | 1 | 0 | 1 | 1 | 1 | 1 | 1 | 1 | 7 |
| Awoke et al | 0 | 1 | 0 | 1 | 1 | 1 | 1 | 1 | 1 | 7 |
| Bekele et al | 0 | 1 | 0 | 1 | 1 | 1 | 1 | 1 | 1 | 7 |
| Bizuayehu et al | 1 | 1 | 1 | 1 | 1 | 1 | 1 | 1 | 1 | 9 |
| Dessie et al | 1 | 1 | 0 | 1 | 1 | 1 | 1 | 1 | 1 | 8 |
| Gashaw et al | 1 | 1 | 1 | 1 | 1 | 1 | 1 | 1 | 1 | 9 |
| Gebissa et al | 0 | 1 | 1 | 1 | 1 | 1 | 1 | 1 | 1 | 8 |
| Mekonen et al | 0 | 1 | 1 | 1 | 1 | 1 | 1 | 1 | 1 | 8 |
| Melaku et al | 1 | 1 | 0 | 1 | 1 | 1 | 1 | 1 | 1 | 8 |
| Misha et al | 1 | 1 | 1 | 1 | 1 | 1 | 1 | 1 | 1 | 9 |
| Motbinor et al | 0 | 1 | 1 | 1 | 1 | 1 | 1 | 1 | 1 | 8 |
| Sahile et al | 1 | 1 | 1 | 1 | 1 | 1 | 1 | 1 | 1 | 9 |
| Tilahun et al | 0 | 1 | 1 | 1 | 1 | 1 | 1 | 1 | 1 | 8 |
| Tilahun et al | 0 | 1 | 1 | 1 | 1 | 1 | 1 | 1 | 1 | 8 |
| Tolera et al | 1 | 1 | 1 | 1 | 1 | 1 | 1 | 1 | 1 | 9 |
| Worku et al | 0 | 1 | 1 | 1 | 1 | 1 | 1 | 1 | 1 | 8 |

1: Yes, 2: No

**S1 Fig: Pooled antimicrobial resistance of P. aeruginosa for amikacin**

**S2 Fig: Pooled antimicrobial resistance of P. aeruginosa for amoxicillin-clavulanic acid**

**

**S3 Fig: Pooled antimicrobial resistance of P. aeruginosa to ampicillin**

**S4 Fig: Pooled antimicrobial resistance of P. aeruginosa to ceftazidime**

**

**S5 Fig: Pooled antimicrobial resistance of P. aeruginosa to ceftriaxone**

**S6 Fig: Pooled antimicrobial resistance of P. aeruginosa to chloramphenicol**

**S7 Fig: Pooled antimicrobial resistance of P. aeruginosa to gentamicin**

**S8 Fig: Pooled antimicrobial resistance of P. aeruginosa to ciprofloxacin**

**S9 Fig: Pooled antimicrobial resistance of P. aeruginosa to Trimethoprime-Sulfamethoxazole**

**S10 Fig: Subgroup analysis of P. aeruginosa associated health-care associated infections by publication year**

**S11 Fig: Subgroup analysis of P. aeruginosa associated healthcare-associated infections by infection type**

**S12 Fig: Subgroup analysis of antimicrobial resistance of P. aeruginosa for amikacin by infection type**

**S13 Fig: Subgroup analysis of antimicrobial resistance of P. aeruginosa for amoxicillin-clavulanic acid by infection type**

**S14 Fig: Subgroup analysis of antimicrobial resistance of P. aeruginosa to ampicillin by infection type**

**S15 Fig: Subgroup analysis of antimicrobial resistance of P. aeruginosa to ceftazidime by infection type**

**S16 Fig: Subgroup analysis of antimicrobial resistance of P. aeruginosa to ceftazidime by publication year**

**S17 Fig: Subgroup analysis of antimicrobial resistance of P. aeruginosa to ceftriaxone by infection type**

**S18 Fig: Subgroup analysis of antimicrobial resistance of P. aeruginosa to ceftriaxone by publication year**

**S19 Fig: Subgroup analysis of antimicrobial resistance of P. aeruginosa to chloramphenicol by infection type**

**S20 Fig: Subgroup analysis of antimicrobial resistance of P. aeruginosa to chloramphenicol by publication year**

**S21 Fig: Subgroup analysis of antimicrobial resistance of P. aeruginosa to ciprofloxacin by infection type**

**S22 Fig: Subgroup analysis of antimicrobial resistance of P. aeruginosa to ciprofloxacin by publication year**

**S23 Fig: Subgroup analysis of antimicrobial resistance of P. aeruginosa to gentamicin by Infection type**

**S24 Fig: Subgroup analysis of antimicrobial resistance of P. aeruginosa to gentamicin by publication year**

**S25 Fig: Subgroup analysis of antimicrobial resistance of P. aeruginosa to Trimethoprim-Sulfamethoxazole by type of infection**

**S26 Fig: Subgroup analysis of antimicrobial resistance P. aeruginosa to meropenem by infection type**

**S27 Fig: Subgroup analysis of antimicrobial resistance P. aeruginosa to meropenem by study year**

**S28 Fig: Subgroup analysis of multi-drug resistant P. aeruginosa by publication year**

**S3 Table: Trim-and-fill analysis for the prevalence of P. aeruginosa associated healthcare-associated infection**

| Studies | Prevalence | 95% CI |
| --- | --- | --- |
| Observed (23) | 4.38 | 3-5.76 |
| Observed + Imputed (23+1) | 4.61 | 3.23-6 |

**S4 Table: Trim-and-fill analysis result of antimicrobial resistance of P. aeruginosa to Trimethoprim-Sulfamethoxazole**

| Studies | Prevalence | 95% CI |
| --- | --- | --- |
| Observed (7) | 75.41 | 58.39-92.43 |
| Observed + Imputed (7+4) | 92.08 | 72.89-111.28 |

**S29 Fig: Funnel plot for AMR of P. aeruginosa to Trimethoprim-Sulfamethoxazole after trim-and-fill analysis**

**S5 Table: Trim-and-fill analysis result of antimicrobial resistance of P. aeruginosa to ceftriaxone**

| Studies | Prevalence | 95% CI |
| --- | --- | --- |
| Observed (22) | 98.72 | 96.39-101.04 |
| Observed + Imputed (22+1) | 99.10 | 96.79-101.41 |

**S30 Fig: Funnel plot for antimicrobial resistance of P. aeruginosa to ceftriaxone after trim-and-fill analysis**

**S6 Table: Trim-and-fill analysis result of antimicrobial resistance of P. aeruginosa to meropenem**

| Studies | Pooled multi-drug resistance | 95% CI |
| --- | --- | --- |
| Observed (12) | 28.636 | 16.35-40.93 |
| Observed + imputed (12+2) | 24.078 | 11.473-36.683 |

**S31 Fig: Funnel plot for antimicrobial resistance of P. aeruginosa to meropenem after trim-and-fill analysis**

**S7 Table: Trim-and-fill analysis on pooled multi-drug resistance profile of P. aeruginosa**

| Studies | Pooled multi-drug resistance | 95% CI |
| --- | --- | --- |
| Observed (15) | 80.05 | 66.25-93.84 |
| Observed + imputed (15+1) | 78.49 | 65.27-91.72 |
